# Chronic respiratory symptoms and pulmonary function status in Ethiopian agricultural workers: a comparative study

**DOI:** 10.1186/s12890-020-1120-3

**Published:** 2020-04-07

**Authors:** Gashaw Garedew Woldeamanuel, Alemu Basazin Mingude, Getachew Yideg Yitbarek, Mitku Mammo Taderegew

**Affiliations:** 1 Department of Biomedical Sciences, School of Medicine, College of Medicine and Health Sciences, Wolkite University, P.O. Box 07, Wolkite, Ethiopia; 2 Department of Nursing, College of Medicine and Health Sciences, Wolkite University, Wolkite, Ethiopia; 3 Department of Biomedical Sciences, College of Health Sciences, Debre Tabor University, Debre Tabor, Ethiopia

**Keywords:** Farmers, Chronic respiratory symptoms, Prevalence, Spirometer, Ethiopia

## Abstract

**Background:**

Work-related respiratory disorders are major contributors to the global burden of respiratory diseases. Agricultural workers are exposed to a number of dusts, which may contribute to the development of respiratory disorders. However, the knowledge about the prevalence of respiratory symptoms and pulmonary function status in African farmers was limited. This study was conducted to assess the prevalence of chronic respiratory symptoms and pulmonary function status of Ethiopian farmers exposed to farming activities.

**Methods:**

A community based comparative cross sectional study was conducted among 288 agricultural workers (farmers) aged 18 to 65 years and 288 control subjects (non-agricultural workers). Data were collected by interviewer administered structured questionnaires adopted from British Medical Research Council respiratory questionnaire and American Thoracic Society Division of Lung Diseases questionnaire. Moreover, all study participants underwent spirometry.

**Results:**

The prevalence of chronic respiratory symptoms was higher in farmers than in controls, with significant difference for cough (20.5% vs. 9.0%, *p* = 0.001) and phlegm (19.1% vs. 9.0%, *p* = 0.001). Chronic respiratory symptoms among farmers were significantly associated with duration of agricultural exposure (*p* = 0.014). The mean values of all spirometric parameters were lower in farmers than in controls with significant difference for FVC, FEV1, FEF_25%_, FEF_50%_, FEF_25–75%_ and PEFR. Moreover, the mean values of all spirometric parameters of farmers have found to be decreased with increased duration of workplace exposure. Lung function test results also showed a higher prevalence of obstructive (15.6% vs. 10.8%, *p* = 0.085) and restrictive type (12.8% vs. 3.1%, *p* <  0.001) of pulmonary function impairment in farmers than in controls. However, the difference was not significant for obstructive type of impairment.

**Conclusion:**

This study indicated that farmers are at high risk for the development of chronic respiratory symptoms and reduced pulmonary function indices. Hence, a comprehensive occupational safety practices are important to maintain the respiratory health of farmers.

## Background

Chronic respiratory symptoms including chronic cough, phlegm, wheezing, shortness of breath, and chest tightness could be the manifestations of chronic respiratory diseases, which are mainly developed as a result of exposure to occupational hazards [1, 2]. Occupational exposures to respiratory hazards remain significant contributors to the global burden of respiratory disease [3] and the burden of occupational respiratory diseases is increasing worldwide [4].

Agriculture is the dominant occupation [5] and one of the most important economic sectors in the world [6]. In developing countries, large proportions (about 80%) of the economically active population are involved in agricultural activities [5]. However, an agricultural activity has been described as a dangerous unhealthy occupation [7]. It is likely that workers in agriculture sector are exposed to a number of potentially hazardous exposures such as pesticides, pollens, gases, dusts, particulates in the ambient air and zoonotic microbes that may contribute to the development of respiratory disorders [8–10]. Moreover, the increasing use of different chemical substances such as pesticides and other agrochemicals further aggravates the risks [11].

Respiratory disease is a widely recognized occupational problem among agricultural workers [12, 13] and it is an important public health problem worldwide [5, 7, 14]. They are known to be at risk for the development of work related respiratory disorders including rhinosinusitis [10, 15], asthma [16, 17], chronic bronchitis [18, 19], chronic obstructive pulmonary disease (COPD) [10, 13] and hypersensitivity pneumonitis [17]. Despite the low prevalence of smoking among farmers, the risk of morbidity and mortality from certain respiratory diseases was high [5]. However, respiratory diseases among farmers are preventable by controlling hazardous exposures and use of personal protective equipments [5, 12].

Respiratory symptoms are common among farmers [15] and exposure to high concentration of dusts during agricultural work leads to high levels of respiratory symptoms [20]. The research in Macedonia found that 26.6% of agricultural workers had chronic respiratory symptoms with higher frequency of chronic cough. A higher prevalence of chronic respiratory symptoms was obtained in agricultural workers with longer duration of workplace exposure [21, 22]. A study on 82 Ethiopian male farmers engaged in the application of pesticides also found a higher prevalence of chronic cough, phlegm and wheeze compared to office workers [23]. Moreover, other studies found lower values of spirometric parameters among agricultural workers compared to office controls [21, 24]. Occupational exposure to agricultural practices is associated with impaired pulmonary function parameters, suggestive of an obstructive or restrictive syndrome [25].

Exposure condition and risk factors for respiratory diseases among farmers were varied in different countries due to climatic conditions or agricultural practices [6, 7]. Despite the early recognition of respiratory hazards in agriculture, the knowledge about the prevalence of respiratory symptoms and pulmonary function status in African farmers working in agricultural sectors was limited. In Ethiopia, respiratory disorder in farmers is likely to be major public health issues, since a large proportion, around 80–85% of Ethiopians are working in agriculture [26]. However, study of respiratory symptoms and pulmonary function parameters in farmers using non farming working subjects as controls is still lacking. Therefore, this study assessed the prevalence of chronic respiratory symptoms and pulmonary function status of Ethiopian farmers exposed to farming activities.

## Methods

### Study design and setting

A community based comparative cross sectional study was conducted to assess the prevalence of chronic respiratory symptoms and lung function status among agricultural workers in Abeshge district, Southern Ethiopia. Abeshge district is found in the southern region of Ethiopia and located 158 km away from Addis Ababa, the capital city of Ethiopia. The study was conducted from April 20 to June 30, 2019.

### Study population and sampling techniques

The source populations were all agricultural workers (farmers) residing in Abeshge district, Southern Ethiopia, whereas the study populations were all agricultural workers and controls in the selected kebeles of the district. A kebele (peasant association) is a smallest administrative unit of Ethiopia that encompasses small communities such as villages. It is part of a woreda (district), which is a part of zone, which in turn are grouped together to form a region that comprise the national state (Ethiopia).

The case group consisted of agricultural workers in the age range between 18 and 65 years, whereas non agricultural workers with no history of occupational exposure to respiratory hazards were selected as a control group. The inclusion criteria for this study were adults between the age of 18 and 65 years, worked as farmers for more than 1 year and those who were volunteered to participate in the study. Smokers (former or current), khat chewers, athletes, pregnant women, those on chronic therapy for any diseases, those who had known cardio-pulmonary disease, other chronic diseases (diabetes, hypertension, renal diseases, etc), or had any contraindication for lung function tests (e.g. history of eye, chest or abdominal surgery, haemoptysis, current respiratory infection and history of pneumothorax, emboli or aneurysms) were excluded from the current study.

The sample size was calculated using double proportion formula by considering 20 and 8% prevalence of cough for case group and control group, respectively [24], 95% confidence interval (CI), 80% power [27–29], farmer to non-farmer ratio of 1:1, design effect of 2 and 90% response rate. Accordingly, 576 participants (288 farmers and 288 non-farmers) who fulfilled the inclusion criteria were recruited in the study.

Multistage cluster sampling procedure was applied to select the study participants. First, four kebeles from the 29 kebeles of Abeshge district were selected using lottery method. Then, in each of the four kebeles, three villages were selected randomly by using lottery method. Finally, all adults lived in the selected villages and those who fulfilled the inclusion criteria were included in the study. Participants of both groups (case group and control group) were selected using the same procedures.

### Operational definitions

Chronic respiratory symptoms were defined as the development of one or more of the symptom (s) of chronic cough, chronic phlegm, chronic wheezing, chronic shortness of breath, and chronic chest tightness which last (s) at least 3 months in 1 year. Detail definitions of each respiratory symptom were published elsewhere [1, 2] and used in this study. The modified Medical Research Council (mMRC) Dyspnoea Scale was used as a measure of dyspnoea severity, ranged from 0 to 4, being 0 indicated no breathlessness and 4 indicated too breathless to leave the house. Moreover, pulmonary function impairment was classified into different categories as obstructive impairment (FEV1/FVC <  0.7), restrictive impairment (FEV1/FVC ≥ 0.7 and FVC < 80% predicted), symptoms only (if the participants had any respiratory symptoms) and no impairment or normal [30–32].

### Data collection procedures

Data were collected by using pre-tested and structured questionnaires adopted from British Medical Research Council (BMRC) respiratory questionnaire [33] and American Thoracic Society Division of Lung Diseases questionnaire (ATS − DLD −78A) [34]. The questionnaire contained different factors such as socio- demographic factors, occupational history, medical history and respiratory symptoms. The data were collected by trained data collectors using face to face interview. After interview, body weight was measured using weighing scale and approximated to the nearest 0.1 kg. Similarly, height was measured by using an erect height measuring device and approximated to 0.1 cm. Then, body mass index (BMI) of the study participants was calculated from their body weight and height using weight in kilogram / (height in meter)^2^ as a formula [35].

A digital portable Spirometer (Spirolab MIR, Italy) was used to measure lung function parameters. All participants underwent spirometry based on the American Thoracic Society and European Respiratory Society (ATS/ERS) guidelines [36]. Before the actual test, calibration of spirometers was done. Moreover, the test procedure was explained to the participants and advised to take rest for at least 5 min. Then after, spirometry was performed in sitting position by trained technicians. A minimum of three acceptable spirometric measurements were performed with adequate rest in between and the best values were used for analysis. The parameters taken during the test included forced vital capacity (FVC), forced expiratory volume in 1 second (FEV_1_), FEV_1_: FVC ratio, forced expiratory flow 25% (FEF_25%_), FEF_50%_, FEF_75%_, FEF_25–75%_ and peak expiratory flow rate (PEFR).

To maintain data quality, intensive training was given to the data collectors and supervisors prior to the actual data collection. The questionnaire was pre-tested on 5% of the actual sample size (5% of 576 subjects or 29 subjects) that were not included in the main survey and necessary corrections were undertaken accordingly. Moreover, spirometer was calibrated and checked before the actual test and all spirometric measurements were performed at a fixed time of the day by using the same instructions for all participants. Any spirometry results with poor quality were not used for analysis.

### Data processing and analysis

The data were checked, coded and entered into Epi-data manager 4.4 and then analysis was made using statistical package for social sciences (SPSS) version 23 software. Descriptive statistics was computed to summarize some characteristics of the study participants. Chi-square (χ2) test and independent sample t-test were used to compare categorical variables and continuous variables of the two groups, respectively. Moreover, binary logistic regression analysis was performed to estimate the association between chronic respiratory symptoms and demographic factors. Variables like sex, BMI, educational status and duration of employment were included in the logistic regression model. Similarly, linear regression analysis was performed to explore associations between spirometric parameters and exposure duration. For all statistical tests, p – value < 0.05 was considered as significant.

## Results

### Socio-demographic characteristics

The research team visited 623 adults in the randomly selected villages. Of these, 324 subjects were farmers and 299 subjects were in the group of controls. However, 23 adults (17 agricultural workers and 6 control subjects) declined participation in the study and 24 adults (19 agricultural workers and 5 control subjects) provided unacceptable spirometry and therefore excluded from the study. A total of 576 (288 agricultural workers and 288 control subjects) individuals were involved in the study. More than half of the respondents were men for both groups [154 (53.5%) agricultural workers and 168 (58.3%) control subjects]. The age of agricultural workers ranged from 25 to 64 years with a mean of 39.35 (± 9.00) years, while for control subjects, it ranged from 25 to 64 years with a mean of 38.06 (± 8.30) years. Regarding to demographic characteristics of the study participants, there was no significant difference between the two examined groups (*p* > 0.05) (Table 1).

**Table 1:** Socio-demographic characteristics of the study participants

Characteristics	Agricultural workers (***n*** = 288)	Control subjects (***n*** = 288)	Total	***p*** - value
**Sex**				
Male	154 (53.5%)	168 (58.3%)	322 (55.9%)	0.240
Female	134 (46.5%)	120 (41.7%)	254 (44.1%)	
*Age (years)	39.35 (9.00)	38.06 (8.30)	38.70 (8.68)	0.074
**Religion**				
Muslim	131 (39.2%)	85 (29.5%)	198 (34.4%)	0.060
Orthodox	144 (50.0%)	167 (58.0%)	311 (54.0%)	
Protestant	22 (7.6%)	30 (10.4%)	52 (9.0%)	
Catholic	9 (3.1%)	6 (2.1%)	15 (2.6%)	
**Marital status**				
Single	27 (9.4%)	45 (15.6%)	72 (12.5%)	0.088
Married	242 (84.0%)	227 (78.8%)	469 (81.4%)	
Divorced	7 (2.4%)	9 (3.1%)	16 (2.8%)	
Widow /widower	12 (4.2%)	7 (2.4%)	19 (3.3%)	
*Duration of -employment (years)	16.54 (9.04)	15.39 (6.76)	15.96 (7.99)	0.085
*Height (meter)	1.62 (0.07)	1.63 (0.05)	1.63 (0.06)	0.118
*Weight (kg)	56.56 (9.18)	57.92 (8.25)	57.4 (8.75)	0.061
*BMI (kg/m^**2**^**)**	21.36 (2.99)	21.66 (2.75)	21.51 (2.87)	0.218

Note: *: Data are presented as mean (standard deviation), *BMI* Body Mass Index

### Prevalence of chronic respiratory symptoms

A total of 168 individuals [108 (37.5%) agricultural workers and 60 (20.8%) control subjects] reported at least one chronic respiratory symptom. The percentage of individuals with at least one chronic respiratory symptom was significantly higher in agricultural workers as compared to control groups (*p* <  0.001). Overall, prevalence of chronic respiratory symptoms was higher in agricultural workers than in controls with statistical significant difference for chronic cough and chronic phlegm (Table 2). According to the mMRC dyspnoea scale, 24 (8.3%) and 18 (6.2%) agricultural workers and 18 (6.2%) and 12 (4.2%) control subjects had dyspnoea score 1 and 2, respectively. None of the study participants had dyspnoea score 3 or 4.

**Table 2:** Prevalence of chronic respiratory symptoms in agricultural workers (n = 288) compared with their controls (*n* = 288)

Symptoms	Agricultural workers ***n*** (%)	Control subjects n (%)	***p***-value
Any respiratory-symptoms	108 (37.5)	60 (20.8)	< 0.001*
Cough	59 (20.5)	26 (9.0)	0.001*
Phlegm	55 (19.1)	26 (9.0)	0.001*
Dyspnoea	42 (14.6)	30 (10.4)	0.131
Wheezing	20 (6.9)	10 (3.5)	0.061
Chest tightness	47 (16.3)	37 (12.8)	0.238

**Note:** Numerical data in * indicates the level of significance (*p* < 0.05)

After adjusting for sex, BMI and educational status in binary logistic regression model, increasing duration of exposure to agricultural activities was associated with an increased likelihood of exhibiting chronic respiratory symptoms (AOR = 1.04, 95% CI [1.01, 1.06], *p* = 0.014) (Table 3). We found a higher prevalence of chronic cough (23.8% vs. 16.8%, *p* = 0.139), chronic phlegm (25.8% vs. 11.7%, *p* = 0.002), chronic shortness of breath (18.5% vs. 10.2%, *p* = 0.046), chronic wheezing (7.3% vs. 6.6%, *p* = 0.811) and chronic chest tightness (22.5% vs. 9.5%, *p* = 0.003) in farmers with duration of exposure ≥15 years than in farmers with duration of exposure < 15 years, respectively. However there was no statistically significant difference for cough and wheezing.

**Table 3:** Chronic respiratory symptoms in relation with some demographic factors and workplace exposure duration among agricultural workers (*n* = 288)

Characteristics	Chronic respiratory symptoms		COR (95%CI)	AOR (95%CI)
	Yes, ***n*** (%)	No, ***n*** (%)		
**Sex**				
Male	56 (36.4)	98 (63.6)	1.00	1.00
Female	52 (38.8)	82 (61.2)	1.11 (0.69–1.79)	1.09 (0.66–1.83)
**BMI (kg/m**^**2**^**)**				
< 18.5	19 (47.5)	21 (52.5)	1.11 (0.38–3.25)	0.94 (0.31–2.84)
18.5–24.9	80 (35.1)	148 (64.9)	0.66 (0.26–1.66)	0.71 (0.28–1.80)
≥ 25	9 (45.0)	11 (55.0)	1.00	1.00
**Educational level**				
Illiterate	68 (41.0)	98 (59.0)	1.80 (0.82–3.98)	1.42 (0.62–3.27)
Primary school	30 (34.9)	56 (65.1)	1.39 (0.59–3.27)	1.41 (0.59–3.35)
≥ Secondary school	10 (27.8)	26 (72.2)	1.00	1.00
≠**Exposure duration (in years)**	18.56 (10.06)	15.36 (8.14)	1.04 (1.01–1.07)	1.04 (1.01–1.06)*

**Note:** Numerical data in * indicates the level of significance (*p* = 0.014), ≠: Data are presented as mean (standard deviation), *BMI* Body Mass Index, *CI* Confidence interval, *1.00* reference group, *COR* Crude odds ratio, *AOR* Adjusted odds ratio, which was adjusted for sex, *BMI*, educational status and exposure duration. Age was not included in the model due to the issue of multicollinearity

### Pulmonary function tests

The mean values of spirometric parameters were lower in agricultural workers as compared to control subjects with significant difference for FVC, FEV1, FEF_25%_, FEF_50%_, FEF_25–75%_ and PEFR. However, mean values of FEV1: FVC ratio and FEF_75%_ were not significantly different between the two groups (Table 4). The percentage predicted values of all spirometric parameters were also lower in agricultural workers compared with their controls (Table 5).

**Table 4:** Actual mean values of spirometric parameters in agricultural workers (*n* = 288) compared with their controls (*n* = 288)

Parameters	Agricultural workers Mean ± SD	Control subjects Mean ± SD	95% CI	***p***-value
FVC (L)	3.11 ± 1. 03	3.34 ± 1.10	− 0.40, − 0.05	0.010*
FEV_1_ (L)	2.53 ± 0.87	2.73 ± 0.82	− 0.34, − 0.06	0.004*
FEV_1_/ FVC (%)	82.43 ± 14.03	83.67 ± 13.42	−3.49, 1.01	0.279
FEF_25%_ (L/s)	8.53 ± 1.94	8.95 ± 1.85	− 0.73, − 0.11	0.008*
FEF_50%_ (L /s)	3.35 ± 1.51	3.64 ± 1.51	− 0.54, − 0.04	0.022*
FEF_75%_ (L/s)	2.20 ± 0.89	2.34 ± 0.95	− 0.29, 0.01	0.076
FEF_25–75%_ (L /s)	3.09 ± 1.37	3.32 ± 1.28	− 0.44, − 0.01	0.047*
PEFR (L/s)	4.48 ± 1.94	5.02 ± 1.95	− 0.85,-0.22	0.001*

**Note:** *CI* Confidence interval of the mean difference, *FVC* Forced Vital Capacity, *FEV1* Forced Expiratory Volume in first second, *FEF* Forced Expiratory Flow, *PEFR* Peak Expiratory Flow Rate, *SD* standard deviation, *L* liter, *S* second, Numerical data in * indicates the level of significance (*p* < 0.05)

**Table 5:** Percentage predicted values of spirometric parameters in agricultural workers (n = 288) compared with their controls (n = 288)

Parameters	Agricultural workers Mean ± SD	Control subjects Mean ± SD	***p***-value
FVC	87.20 ± 30.18	95.62 ± 33.64	0.002*
FEV_1_	79.14 ± 28.08	85.66 ± 27.05	0.005*
FEV_1_/ FVC	95.43 ± 24.89	97.18 ± 24.77	0.398
FEF_25%_	98.90 ± 20.83	104.29 ± 22.41	0.003*
FEF_50%_	87.48 ± 28.59	92.90 ± 28.50	0.023*
FEF_75%_	93.79 ± 41.14	97.55 ± 37.89	0.254
FEF_25–75%_	81.69 ± 30.76	86.70 ± 30.92	0.052
PEFR	55.37 ± 19.21	72.18 ± 20.39	< 0.001*

**Note:** *FVC* Forced Vital Capacity, *FEV1* Forced Expiratory Volume in first second, *FEF* Forced Expiratory Flow, *PEFR* Peak Expiratory Flow Rate, *SD* standard deviation, Numerical data in * indicates the level of significance (*p* < 0.05)

Simple linear regression analysis showed that duration of workplace exposure among agricultural workers was significantly associated with FVC, FEV_1_, FEF_25%_, FEF_50%_, FEF_75%_, FEF_25–75%_ and PEFR. After adjusting for sex, educational status and BMI in a multiple linear regression model, an increased duration of workplace exposure remained significantly associated with decreased values of these spirometric parameters (Table 6).

**Table 6:** Univariate simple and multiple linear regression model examining the association between spirometric parameters and duration of workplace exposure (years) in agricultural workers (*n* = 288)

Parameters	Simple linear regression model			Multiple linear regression model		
	Beta coefficient	95% CI	***p***-value	Beta coefficient	95% CI	***p***- value
FVC (L)	−0.023	− 0.037, − 0.010	< 0.001*	−0.019	− 0.031, − 0.007	0.003*
FEV_1_ (L)	− 0.020	−0.031, − 0.009	< 0.001*	−0.020	− 0.030, − 0.010	< 0.001*
FEV_1_/ FVC (%)	−0.071	− 0.251, 0.110	0.442	− 0.165	−0.355, 0.024	0.087
FEF_25%_(L/s)	−0.033	−0.057, − 0.008	0.010*	−0.039	− 0.064, − 0.014	0.003*
FEF_50%_(L/s)	− 0.033	−0.052, − 0.014	0.001*	−0.037	− 0.056, − 0.017	< 0.001*
FEF_75%_ (L/s)	−0.018	− 0.029, − 0.007	0.002*	−0.020	− 0.031, − 0.008	0.001*
FEF_25–75%_ (L/s)	−0.028	− 0.045, − 0.011	0.002*	−0.032	− 0.049, − 0.014	< 0.001*
PEFR (L/s)	−0.025	− 0.050, 0.000	0.046*	− 0.031	−0.056, − 0.006	0.015*

**Note:** *CI* Confidence interval for beta coefficient, *FVC* Forced vital capacity, *FEV1* Forced Expiratory Volume in first second, *FEF* Forced expiratory flow, *PEFR* Peak Expiratory Flow Rate, *L* liter, *S* second, Numerical data in * indicates the level of significance (*p* < 0.05). A multiple regression model was controlled for sex, educational status and BMI. Age was not included in the model due to the issue of multicollinearity

### Pulmonary function impairments

**Fig. 1:**
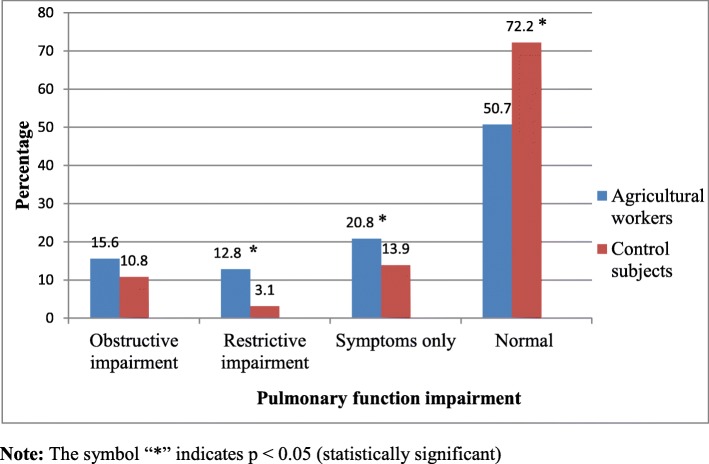
Percentages of agricultural workers (*n* = 288) and control subjects (*n* = 288) stratified by pulmonary function impairment

In this study, obstructive and restrictive pattern of pulmonary function impairment was found in 13.2% (76/576) and 8% (46/576) of the participants, respectively. Among agricultural workers, 146 (50.7%) adults had normal pulmonary function, 45 (15.6%) had obstructive impairment, 37 (13.8%) had restrictive impairment and 60 (20.8%) had respiratory symptoms only. Overall, the prevalence of pulmonary function impairment was higher in agricultural workers than in controls with statistical significant difference for restrictive impairment and symptoms only (*p* <  0.001) (Fig. 1).

## Discussion

Environmental working conditions, notably exposure to respiratory hazards, have been associated with increases in respiratory disorders [20]. Farmers could be exposed to various potentially hazardous exposures that cause respiratory symptoms and ventilatory capacity impairment [37]. Despite the early recognition of respiratory problems in farm workers, the burden of chronic respiratory symptoms and pulmonary function status among rural Ethiopian farmers are largely unknown. To the best of our knowledge, this is the first spirometry and community based study that compared the prevalence of chronic respiratory symptoms and pulmonary function parameters between farmers and control subjects in Ethiopia.

In the present study, the prevalence of overall chronic respiratory symptoms among farmers and control subjects was 37.5 and 20.8%, respectively. In comparison to our result, the research conducted in Macedonia [21] showed a lower prevalence of respiratory symptoms among farmers (26.6%) and office workers (19.1%). Another study by Stoleski et al. [24] found a 29.9% prevalence of overall chronic respiratory symptoms in agricultural workers. A study conducted in Europe reported that frequency of respiratory symptoms in farmers ranged from 25 to 35% [38]. In our study, all participants in the group of farmers did not use personal safety equipments during agricultural practices that could lead to a higher prevalence of respiratory symptoms. Moreover, the differences in the current findings from other studies could be due to variations in farming practices, methodological differences and heterogeneity of study populations. Overall, in line with our findings, an increased risk of developing respiratory symptoms in agricultural workers compared to other occupations was reported in many studies [7, 15, 24, 37, 39].

This study also showed that farmers had an increased prevalence of all respiratory symptoms with statistical significant difference for chronic cough and chronic phlegm than the control subjects. Chronic cough and chronic phlegm were present in 20.5 and 19.1% of the farmers, respectively. In agreement with the current finding, a study conducted among agricultural workers in Macedonia [24] reported that cough was present in 20% of agricultural workers, whereas a lower prevalence of cough (8%) was observed in the office workers. A similar study conducted in Zabul city, Iran [40] also reported that the commonest breathing complaints of the farmers were shortness of breath with 46% and coughing with 40%. Other study has indicated that the prevalence of work-related respiratory symptoms such as wheeze, cough and dyspnoea is unusually high among farmers (23–50%) [12]. Moreover, a study conducted in European animal farmers found that the prevalence of chronic phlegm among farmers was significantly higher than in the general population (9.4% versus 7.5%) [7]. These differences in the frequency of symptoms might be due to a multiplicity of exposures and exposure circumstances in agricultural workers that can result in varying degrees and types of respiratory symptoms [5, 41].

In the present study, duration of workplace exposure was associated with respiratory symptoms. The risk for development of work-related respiratory symptoms increased with the number of years worked in agricultural activities, an observation that agrees with previous reports in agricultural workers (20–22, 24). Chronic exposure to agriculture related dust is implicated in respiratory disease development and its severity [5].

In this study, the mean values of all spirometric parameters were found to be lower among agricultural workers as compared to control subjects with statistical significant difference for FVC, FEV1, FEF_25%_, FEF_50%_, FEF_25–75%_ and PEFR. In line with our finding, a similar study in Iran showed that average amounts of all spirometer parameters of the farmers were significantly less than that of the non-farmers [40]. Ventilatory capacity tests were significantly reduced for farm-workers [37]. Lung function test results of other study also indicated that mean values of all spirometric parameters were lower in agricultural workers as compared to office workers with statistical significance for FEF_50%_, FEF_75%_ and FEF_25–75%_ [24]. In agreement with the result of other studies [24, 42], the present study found that increased duration of workplace exposure was associated with decreased pulmonary function parameters.

The present study also assessed the types of pulmonary function impairment observed among the study participants. Accordingly, obstructive and restrictive ventilatory pattern was observed in 15.6 and 12.8% of the farmers, respectively. This finding is lower in comparison to other study [40], which reported that 22 and 38% of the farmers contracted obstructive and restrictive ventilatory pattern, respectively. This discrepancy from our finding may be due to differences in climate, working conditions and study populations. In our study, the prevalence of obstructive and restrictive type of impairment was higher among farmers as compared to controls with statistical significance for restrictive pattern. There have also been previous reports suggesting a higher risk of obstructive or restrictive patterns in farmers compared to unexposed subjects [24, 25, 43].

The present study has some limitations. First, all farmers in our study area were involved in a similar type of agricultural activities such as cultivation, growing, harvesting and processing of crops and also breeding, raising and caring of animals. Thus, the effect of each agricultural activity on respiratory symptoms and pulmonary function parameters were not assessed. Second, the components of the dusts including the nature and type of the dust were not characterized. Results are reported without any specification of the exposures. Third, we do not have information about second-hand smoke or in-home smoke exposure between the two groups that may confound the effect of agricultural work exposure on respiratory symptoms. Fourth, the study was based on self- reported respiratory symptoms which may be prone to recall bias. However, both farmers and control groups were asked similar questions by a well- trained interviewer. Fifth, skin prick testing was not performed. Finally, this study was cross sectional that couldn’t determine the causal and effect associations of workplace exposure with respiratory symptoms and pulmonary function parameters. Despite these limitations, this study provides valuable information about the burden of chronic respiratory symptoms and pulmonary function impairments in Ethiopian farmers.

## Conclusion

In this study, we found a higher prevalence of chronic respiratory symptoms with significant difference for chronic cough and chronic phlegm, as well as lower mean values of all spirometric parameters with significant difference for FVC, FEV1, FEF_25%_, FEF_50%_, FEF_25–75%_ and PEF in agricultural workers than in controls. The presence of chronic respiratory symptoms was significantly associated with duration of agricultural exposure and spirometric parameters of agricultural workers have found to be decreased with increased duration of workplace exposure. Hence, designing a comprehensive occupational health education programs, safety practices and using personal protective equipments are important to maintain the respiratory health of agricultural workers and to prevent its adverse effects.

## Data Availability

The datasets used and/or analyzed during the current study are available from the corresponding author on reasonable request.

## Abbreviations

FEF: Forced expiratory flow
FEV1: Forced expiratory volume in 1 s
FVC: Forced vital capacity
mMRC: Modified Medical Research Council
PEFR: Peak Expiratory Flow Rate
SD: Standard deviation
